# Severe adenovirus pneumonia complicated by acute respiratory distress syndrome in immunocompetent patients: a case report and literature review

**DOI:** 10.3389/fmed.2025.1524783

**Published:** 2025-03-17

**Authors:** Lei Zhang, Yuxin Guo, Xudong Wang, Wei Gai, Lina Liu

**Affiliations:** ^1^Department of Emergency Medicine, Aerospace Center Hospital, Beijing, China; ^2^Willingmed Technology (Beijing) Co., Ltd., Beijing, China

**Keywords:** human adenovirus, pneumonia, acute respiratory distress syndrome, metagenomic next-generation sequencing, treatment

## Abstract

**Background:**

Human adenovirus (HAdV) is one of the most important pathogens detected in acute respiratory illness in pediatric and immunocompromised patients, but it is relatively rare to develop severe pneumonia in immunocompetent patients. We analyzed the clinical features, as well as the diagnosis and treatment processes, to provide a reference for clinical practice.

**Case presentation:**

We report a case of severe pneumonia caused by HAdV, complicated by acute respiratory distress syndrome (ARDS), in an immunocompetent patient with no underlying conditions. Chest computed tomography (CT) revealed consolidation in the right lower lung. Conventional microbial tests were negative, but metagenomic next-generation sequencing (mNGS) identified a large number of HAdV sequences in blood and sputum. Together with the clinical symptoms, this confirmed the diagnosis of severe pneumonia caused by HAdV. The patient was discharged after timely treatment with cidofovir.

**Conclusion:**

In our study, we described a rare case of severe pneumonia caused by HAdV, complicated by ARDS, in an immunocompetent patient. mNGS proves to be an effective diagnostic tool for guiding treatment decisions.

## 1 Introduction

Human adenovirus (HAdV) is a widely distributed pathogen in the Adenoviridae family. It is a non-envelope, double-stranded DNA virus (1). HAdV can lead to diffuse multi-organ disease in young children and immunocompromised patients, and in severe cases can lead to death, though it is rare in immunocompetent adults (2). HAdV can cause multi-organ infections of the respiratory tract, conjunctiva, gastrointestinal tract, and urinary tract (3). Several HAdV subtypes have been associated with respiratory tract infections, among which the human adenovirus B subtype (HAdV B) is one of the main pathogens responsible for acute respiratory infections, with cough and tonsillar enlargement being the main symptoms (4). Severe viral infections can lead to respiratory failure, which may rapidly progress to acute respiratory distress syndrome (ARDS) (5). The survival rate of patients with ARDS caused by HAdV pneumonia has been reported to be very low (6). Severe ARDS combined with HAdV pneumonia is also a concern in immunocompetent individuals.

Early identification of the etiology in critically ill patients is crucial for clinicians, and clear results can help doctors implement targeted interventions in a timely manner, thereby improving the prognosis of patients. In current clinical microbiology laboratories, reverse transcriptase-polymerase chain reaction (RT-PCR) and serological antigen-antibody tests are commonly used for rapid detection of the virus. However, due to the influence of viral copy number, conventional viral tests are often prone to false-negative results (7). In recent years, metagenomics next-generation sequencing (mNGS) has played an important role in the etiological diagnosis of various infection, as a rapidly developing technology for pathogen detection (8). Several studies have shown that mNGS can detect pathogens with negative results from conventional methods, showing higher sensitivity than conventional methods (9). mNGS is also increasingly used to diagnose pathogens in difficult and critically ill patients.

Here, we report a case of ARDS caused by severe HAdV pneumonia and use mNGS to identify a clinically relevant pathogen. We also review previous studies that focusing on the diagnosis and treatment of ARDS caused by severe HAdV pneumonia.

## 2 Case presentation

**FIGURE 1:**
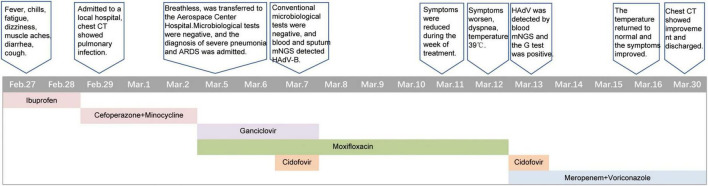
Clinical course of the patient.

An 18-year-old man presented with a fever up to 40°C on 27 February 2024 with chills, fatigue, muscle aches, dizziness, diarrhea, and cough symptoms. He self-medicated with ibuprofen, but his symptoms did not improve. On February 29, 2024, a chest computed tomography (CT) scan performed at a local hospital showed a lung infection. He was treated with cefoperazone-sulbactam in combination with minocycline for 3 days, but his symptoms persisted. On March 4, 2024, he developed dyspnea, which worsened with activity and remained unresolved. He was transferred to the emergency department of our hospital on March 5, 2024. The patient had been previously healthy (Figure 1).

**FIGURE 2:**
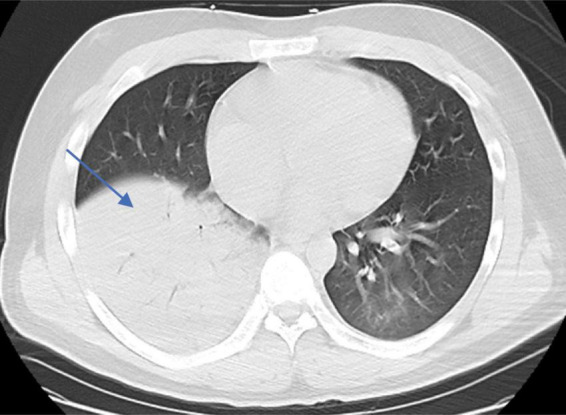
Chest CT imaging features of the patient upon admission (patchy hyperdense lesions in the left lower lobe and consolidation in the lower lobe of the right lung).

On admission (day 1), the physical examination revealed a temperature of 39°C, a pulse of 118 beats/min, a respiratory rate of 35 breaths/min, and blood pressure of 102/61 mmHg. There was no palpable swelling of superficial lymph nodes, breath sounds were clear in both lungs, crackles were present in the right lower lung, the heart rhythm was regular, the abdomen was flat with no palpable mass, Murphy’s sign was negative, and there was no edema in the lower extremities. Laboratory tests showed a white blood cell count (WBC) of 3.95 × 10^9^/L, lymphocyte percentage (LY%) of 16.8%, neutrophil percentage (NE%) of 81.2%, hemoglobin (Hb) of 102 g/L, platelet count (PLT) of 63 × 10^9^/L, serum amyloid A (SAA) > 300 mg/L, interleukin-6 (IL-6) of 20 pg/mL, and C-reactive protein (CRP) of 62.8 mg/L, procalcitonin (PCT) of 3.54 ng/mL. Arterial blood gas (ABG) analysis showed a pH value of 7.44, PO2 of 45 mmHg, PCO2 of 28 mmHg, aspartate aminotransferase of 84.7 U/L, and lactate dehydrogenase of 480.90 U/L. The serologic tests for respiratory pathogens (*Legionella pneumophila*, *Mycoplasma pneumoniae*, *Chlamydia pneumoniae*, *respiratory syncytial virus*, HAdV, and *coxsackievirus*) and HIV were negative. RT-PCR tests for SARS-CoV-2, *influenza virus*, *Epstein-Barr virus*, and *cytomegalovirus* were also negative (Table 1). Chest CT showed multiple patchy hyperdensities with blurred margins in the left lower lobe. The lower lobe of the right lung was a consolidation opacity. The initial diagnosis on admission was severe pneumonia and ARDS (Figure 2).

**TABLE 1:** Clinical indicators and laboratory test results during the diagnosis and treatment process of the patient.

Date	Temperature (C°)	WBC (10^9^/L)	LY%	NE%	CRP (mg/L)	PCT (ng/mL)	IL-6 (pg/mL)	Microbiological test results
2024/3/5	39	3.95	16.8	81.2	62.8	3.54	20	Serology for respiratory pathogens, including Legionella pneumophila (−), Mycoplasma pneumoniae (−), Chlamydia pneumoniae (−), respiratory syncytial virus (−), HIV (−), HAdV (−), and coxsackievirus (−). RT-PCR tests, including SARS-CoV-2 (−), influenza virus (−), Epstein-Barr virus (−), and cytomegalovirus (−).
2024/3/7	39.2	6.02	7.4	90	—	—	—	Blood and sputum mNGS: HAdV (+) Culture (−) GM tests (−)
2024/3/11	37.5	3.8	13	84	37	0.7	18	
2024/3/13	39	5.3	6.6	93	62	1.2	49	Blood mNGS: HAdV (+) G tests (+)
2024/3/15	38	5.8	10.8	88.5	28	0.45	4.6	
2024/3/16	37.4	5.8	12.5	83.2	10	0.36	11.2	
2024/3/23	37.2	8.2	32	73.2	10	0.25	2	

WBC, white blood cell; LY%, lymphocyte percentage; NE%, neutrophil percentage; CRP, C-reactive protein; PCT, procalcitonin; IL-6, interleukin-6.

**FIGURE 3:**
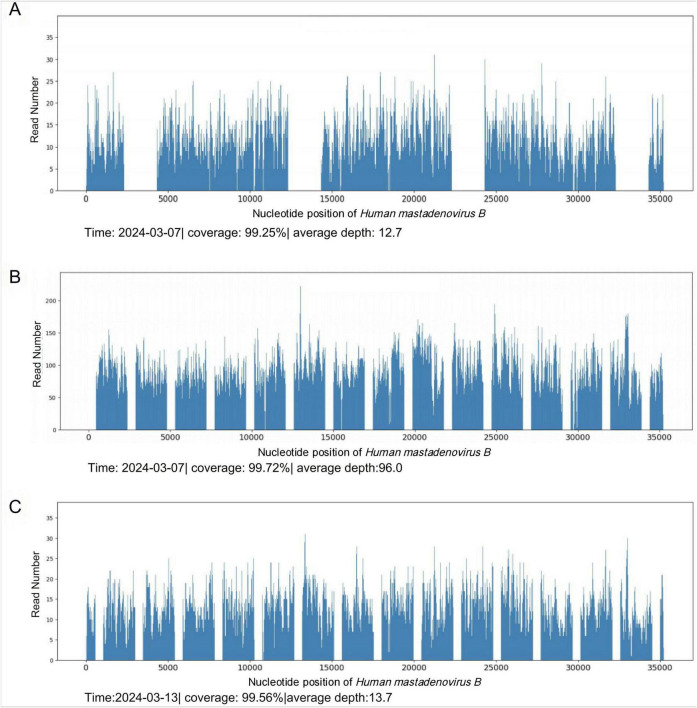
mNGS results. **(A)** HAdV detected by blood mNGS on March 7, 2024. **(B)** HAdV detected by sputum mNGS on March 7, 2024. **(C)** HAdV detected by mNGS in blood on March 13, 2024.

**FIGURE 4:**
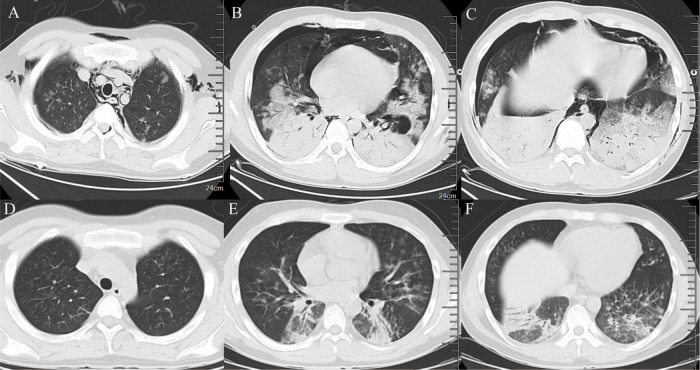
Imaging features of chest CT. **(A–C)** Chest CT on March 12, 2024 (consolidation of both lungs, increased effusion, spontaneous pneumothorax in the right lung, mediastinal emphysema, subcutaneous emphysema). **(D–F)** Chest CT on March 30, 2024 (spontaneous pneumothorax and subcutaneous emphysema disappeared, and pulmonary effusion was significantly reduced).

To alleviate acute respiratory distress, he received oxygen via an oxygen storage mask. Moxifloxacin (0.4 g, qd) was combined with ganciclovir (0.4 g, q12 h) for empiric anti-infective therapy. Methylprednisolone sodium succinate (80 mg, qd) and intravenous human immunoglobulin (20 g, qd) were used concomitantly for anti-inflammatory and immunomodulatory purposes. To determine the cause, blood and sputum samples were collected on March 6, 2024 (the day after admission), Some of these samples were sent to the hospital’s microbiology laboratory for conventional culture and (1, 3) -beta-D-glucan and galactomannan (G/GM) testing, while the other part was sent to Willingmed Technology (Beijing) Co., Ltd. for mNGS testing. The mNGS procedure followed the previous report (10). The next day, mNGS of blood and sputum detected a high sequence number of HAdV B positive (13820 and 77838 reads), and the results of conventional culture and GM tests were negative (Figures 3A, B). Based on the clinical symptoms and microbiological test results, the treatment regimen was changed to moxifloxacin (0.4 g, qd) combined with cidofovir (5 mg/kg, qw) for antiviral therapy. Cidofovir was used concurrently with the recommended dose of probenecid to reduce renal toxicity. At the same time, the dose of methylprednisolone sodium succinate (3 mg/kg, qd) was adjusted for three consecutive days of pulse therapy, after which the dose was gradually reduced, and a noninvasive ventilator was used for assisted ventilation. Within a week of admission, the patient’s symptoms improved, his body temperature gradually decreased, the oxygenation index fluctuated between 81 and 102 mmHg, and his leukocytes, platelets, and procalcitonin returned to normal. However, the proportion of neutrophils remained slightly elevated (84%). On March 12, 2024, the patient’s symptoms worsened again, he developed dyspnea, his body temperature rose to 39°C and his oxygenation index dropped to 62 mmHg. We conducted further analysis of the etiology of exacerbations. A chest CT scan on the same day showed increased effusion from both lungs, spontaneous pneumothorax in the right lung, mediastinal and subcutaneous emphysema (Figures 4A–C). On the same day, we collected blood samples again for mNGS testing. On March 13, 2024, the mNGS results still showed a high load of adenovirus (20140 reads) (Figure 3C). Laboratory tests revealed an elevated PCT level of 1.2 ng/mL, a normal WBC count, an elevated NE% of 93%, and a positive result of G test. Based on the patient’s symptoms and laboratory tests, we considered co-infection with viruses, bacteria, and fungi. Patients received a second dose of cidofovir antiviral therapy in combination with probenecid to reduce renal toxicity. Moxifloxacin was discontinued, and meropenem (0.5 g, q8 h) and oral voriconazole (200 mg, q12 h) were added for anti-infective therapy. Fluid restriction, protein supplementation, diuresis to reduce pulmonary effusion, antipyretic, and nutritional supportive therapy were implemented. After adjusting the treatment, the patient’s peripheral oxygen saturation was maintained at above 90% with the support of high-flow nasal oxygen, and the body temperature returned to normal on March 16, 2024. The patient’s symptoms gradually improved. On March 30, 2024, a chest CT scan showed that spontaneous pneumothorax and subcutaneous emphysema had resolved, and pulmonary effusion was significantly reduced (Figures 4D–F). Blood gas analysis showed an oxygenation index of 430, indicating good recovery, and the patient was successfully discharged.

## 3 Discussion and conclusions

HAdV infection is considered an important source of morbidity and mortality in humans and affecting patients worldwide and of all ages (11). Previous studies have also shown a high mortality rate (41%) in cases of adenovirus pneumonia complicated by ARDS (12). Different HAdV species exhibit a preference for specific organs, with HAdV B usually causing acute respiratory infections. However, HAdV is rare in immunocompetent hosts. We searched the PubMed database for the keywords “human adenovirus,” “ARDS,” and “severe pneumonia” from 2009 to 2024, and extracted relevant case data from these case reports based on keyword screening. Although patients in some cohort studies met our inclusion criteria, their individual clinical information could not be identified and, therefore, was excluded from the literature review. A total of 10 cases of severe pneumonia complicated by ARDS caused by HAdV showing that severe pneumonia complicated with ARDS caused by HAdV is rare (Table 2). Of the 10 patients, their ages ranged from 9 months to 53 years; 5 patients had underlying conditions; 6 cases were initially febrile; specific subtypes of HAdV were identified in 5 patients; positive HAdV results were detected by PCR in all patients. Treatment with cidofovir, oseltamivir and ganciclovir were the mainstay of targeted antiviral therapy. The prognosis was poor for most cases, with six deaths. Cases of HAdV-related severe pneumonia complicated by ARDS in immunocompetent adults have been reported less frequently both domestically and internationally, particularly when non-PCR methods were used.

**TABLE 2:** Reported cases of severe pneumonia with HAdV complicated with ARDS.

Case no.	Age	Manifestation	Underlying disease	Comorbidity	HAdV type	Diagnosis mode	Therapeutic drugs	Outcome
1 (28)	9 months	Symptoms of acute bronchitis,	—	ARDS	HAdV-7	RT-PCR	Vancomycin, ceftazidime, oseltamivir	Survival
2 (29)	36	fever, cough	Arthritis	ARDS	HAdV-4	PCR	Piperacillin, linezolid, cidofovir	Survival
3 (30)	35	Symptoms of lower respiratory tract infection	Rheumatoid arthritis	ARDS	HAdV-B21	RT-PCR	Piperacillin, meropenem, vancomycin, fluconazole, acyclovir, cidofovir	Death
4 (30)	53	Shortness of breath/worsening distress.	Epilepsy, chronic hepatitis C.	ARDS	HAdV-B21	PCR	Piperacillin, cefepime, metronidazole	Death
5 (31)	33	Fever, cough, shortness of breath	Chromosome 6P deletion syndrome	ARDS	HAdV	PCR	Piperacillin, vancomycin, amphotericin, cidofovir	Death
6 (32)	22	Fever, cough and sputum production	Previously healthy	ARDS	HAdV	Multiplex PCR	Oseltamivir, ticarcillin, azithromycin, cidofovir	Survival
7 (32)	21	Fever, cough, dyspnea, diarrhea	Previously healthy	ARDS	HAdV	Multiplex PCR	Oseltamivir, Doripenem, Tecoplan, Ganciclovir, Cidofovir	Death
8 (32)	51	Cough and sputum production	Rheumatoid arthritis	ARDS	HAdV	Multiplex PCR	Cefepime, tecoplan, trimethoprim/sulfamethoxazole, oseltamivir, cidofovir	Recurrence
9 (33)	35	Fever	—	ARDS, Septic shock, hypoxemia	HAdV-11	RT-PCR	Ganciclovir	Death
10 (13)	29	Fever, cough and sputum	Previously healthy	ARDS, Septic shock, renal insufficiency, abnormal hepatic function,	HAdV	PCR	Meropenem, moxifloxacin, linezolid, ribavirin and arbidol	Death

In our study, the patient was a young man who had been previously in good health. Our literature review and previous studies also found that younger men have a higher incidence of severe adenovirus pneumonia (13, 14). Common manifestations of HAdV infection include high fever, cough, diarrhea, and conjunctivitis. In addition to conjunctivitis, these symptoms were also present in the patients in this study (15). A previous cohort study also found that immunocompetent patients with severe adenovirus pneumonia had normal or low WBC counts and elevated NE%. The patient in this study exhibited similar clinical characteristics (16). Patients with HAdV complicated by ARDS often exhibit a lower oxygenation index and SpO2 levels and are more susceptible to bilateral lung consolidation, particularly in the first 2 weeks of illness (13, 17, 18). The major radiographic abnormalities of adenovirus infection are predominantly focal or lobar consolidation, which makes it difficult to distinguish them from bacterial pneumonia, potentially leading to misdiagnosis and unnecessary antibiotic therapy (16). Previous reports of worsening respiratory performance in immunocompetent patients with HAdV complicated by ARDS have shown similar symptom in our case, and we believe that the worsening respiratory situation may reflect the early disease progression of adenovirus infection in these patients (19).

The patient in this study were eventually diagnosed with HAdV B, the predominant HAdV species associated with adult respiratory infections in China (16). Isolated cultures of HAdV can be performed from any specimen type, but usually take longer to obtain results. Serologic testing and PCR are considered the gold standard for viral infections, but both viral load and sample collection procedures in the early stages of infection can lead to false-negative results (20). Previous studies have typically used multiplex real-time PCR to screen positive samples for HAdV, followed by further serotyping when positive results are obtained (16). The complexity of the process also delays obtaining results. mNGS is a promising tool for rapid, culture-free pathogen detection and molecular epidemiology. mNGS has proven successful in multiple previous studies of HAdV epidemiology (21). Multiple studies have demonstrated the applicability of mNGS in the early diagnosis of infection (22). In our case, the patient had a negative adenovirus serology test, but HAdV B was detected by blood and sputum mNGS. The results of mNGS were obtained within 24 h. Due to the unidentified pathogen, the patient received empiric antimicrobial therapy. When mNGS detected human adenovirus, he immediately received targeted antiviral therapy, and his symptoms improved quickly. Therefore, mNGS can help select a precise anti-infective treatment regimen. While mNGS has high sensitivity, its specificity may be affected by contamination or colonization, potentially leading to false positives. In addition, the broad spectrum of mNGS assays makes interpreting the results complex. Clinicians still need to make a comprehensive assessment based on clinical manifestations, imaging findings, laboratory tests, and other relevant information. Furthermore, the high cost of mNGS, compared to traditional microbial testing, may limit its broader application.

There are currently no approved antiviral drugs for the treatment of HAdV pneumonia, although cidofovir and oseltamivir are often used in the management of HAdV infection (16). Cidofovir may be effective, particularly in immunocompetent patients, but clinical data on its efficacy in HAdV pneumonia are limited (23). Adjunctive corticosteroids have been used in several studies to treat HAdV pneumonia (15). Some meta-analyses have shown a good safety profile for corticosteroids in patients with severe CAP (24, 25). Pulsed methylprednisolone treatment led to an improvement in the patient’s respiratory distress (26). In our study, antiviral treatment was combined with pulse therapy using methylprednisolone sodium succinate, and patient showed improvement in symptoms. Several studies have recommended early anti-infective therapy with cidofovir for respiratory failure due to HAdV pneumonia (23, 27). In our study, the patient initially received empiric antimicrobial therapy, but his symptoms did not improve. However, when the patient was given cidofovir as antiviral therapy at a dose of 5 mg/kg per week, his symptoms improved.

In conclusion, our study describes a rare case in which mNGS detected severe pneumonia caused by HAdV in immunocompetent patient with ARDS, who recovered after targeted antiviral therapy. As a novel clinical detection method, mNGS is helpful for the timely diagnosis and targeted therapy of HAdV infections.

## Data Availability

The data presented in the study are deposited in the SRA (https://www.ncbi.nlm.nih.gov/bioproject/PRJNA1232331) repository, accession number PRJNA1232331.
